# Oncology care providers’ awareness and practice related to physical activity promotion for breast cancer survivors and barriers and facilitators to such promotion: a nationwide cross-sectional web-based survey

**DOI:** 10.1007/s00520-021-06706-8

**Published:** 2021-12-01

**Authors:** Yoichi Shimizu, Katsunori Tsuji, Eisuke Ochi, Ryo Okubo, Aya Kuchiba, Taichi Shimazu, Noriatsu Tatematsu, Naomi Sakurai, Hiroji Iwata, Yutaka J. Matsuoka

**Affiliations:** 1 Division of Health Care Research, Center for Public Health Sciences, National Cancer Center Japan, 5-1-1 Tsukiji, Chuo-ku, Tokyo, 104-0045 Japan; 2 Department of Nursing, National Cancer Center Hospital, Tokyo, Japan; 3 Faculty of Bioscience and Applied Chemistry, Hosei University, Tokyo, Japan; 4 Division of Biostatistical Research, Center for Public Health Sciences/Biostatistics Division, Center for Research Administration and Support, National Cancer Center Japan, Tokyo, Japan; 5 Graduate School of Health Innovation, Kanagawa University of Human Services, Kanagawa, Japan; 6 Division of Behavioral Sciences, Center for Public Health Sciences, National Cancer Center Japan, Tokyo, Japan; 7 Department of Integrated Health Sciences, Graduate School of Medicine, Nagoya University, Nagoya, Japan; 8 Cancer Solutions Inc, Tokyo, Japan; 9 Department of Breast Oncology, Aichi Cancer Center Hospital, Nagoya, Japan; 10 Lifestyle Medicine, Cooperative Graduate Program, The Jikei University Graduate School of Medicine, Tokyo, Japan

**Keywords:** Physical activity, Breast cancer, Oncology care providers, Exercise implementation

## Abstract

**Purpose:**

A known barrier to getting breast cancer survivors (BCSs) to engage in habitual exercise is a lack of information on recommended physical activity levels provided to them by oncology care providers (OCPs). However, the actual situation in Japan remains unclear. This study sought to clarify OCPs’ awareness and practice related to Japan’s physical activity recommendation for BCSs and to ascertain barriers to routine information provision.

**Methods:**

We conducted a web-based survey involving members of the Japanese Breast Cancer Society (JBCS) and the Japanese Association of Cancer Rehabilitation between Dec. 2018 and Feb. 2019.

**Results:**

Of 10,830 members, 1,029 (9.5%) responded. Only 19.1% were aware of the details of the JBCS physical activity recommendation, and only 21.2% routinely provided physical activity information to BCSs. Factors related to being aware of the recommendation details were 1) availability of the guidelines, 2) experience reading relevant parts of the guidelines, and 3) involvement in multidisciplinary team case meetings. Barriers to routine information provision were 1) absence of perceived work responsibility, 2) underestimation of survivors’ needs, 3) lack of resources, 4) lack of self-efficacy about the recommendation, and 5) poor knowledge of the recommendation.

**Conclusions:**

Only one fifth of the OCPs routinely provided physical activity information. Barriers to provision were poor awareness, self-efficacy, and attitudes and unavailable resources. The physical activity recommendation needs to be disseminated to all OCPs and an information delivery system needs to be established for BCSs to receive appropriate information and support to promote their engagement in habitual physical activity.

**Supplementary Information:**

The online version contains supplementary material available at 10.1007/s00520-021-06706-8.

## Introduction

Maintaining high physical activity levels is known to play a role in extending the healthy lifespan of breast cancer survivors (BCSs) and in improving their health-related quality of life [1–5]. Accordingly, the American Cancer Society (ACS)/American Society of Clinical Oncology (ASCO) Breast Cancer Survivorship Guideline and the Japan Breast Cancer Society (JBCS) Clinical Practice Guidelines for systemic treatment of breast cancer strongly recommend that survivors maintain high physical activity levels [5–7]. The ACS/ASCO guideline recommends 150 min or more per week of moderate-intensity physical activity (e.g., moderate walking or light jogging sufficient to induce sweating) or 75 min or more per week of vigorous physical activity (e.g., jogging or resistance training) [6]. The JBCS guidelines recommend 60 min or more per week of moderate-to-vigorous intensity physical activity in addition to general physical activity in daily life [5, 7]. Despite such recommendations, 54–67% of BCSs remain physically inactive [8–10].

One of the barriers to maintaining high physical activity levels that patients report is a lack of information on physical activity provided by their oncologist [11–13]. This is despite cancer survivors having shown a strong preference for receiving such information from their oncologist [14, 15]. Several studies have reported that oncologists’ recommendations on exercise, or in combination with other interventions, increased patients’ physical activity levels [16–18], and yet most oncologists still do not recommend physical activity to their patients, for various reasons including lack of time, being unclear about specific exercises to recommend, concerns about the effectiveness of exercise, patient safety, and poor knowledge about exercise [19–21]. Therefore, multidisciplinary team members should share the role of discussing exercise recommendations with cancer survivors to increase their physical activity levels [21].

Taking a multidisciplinary team approach to promoting physical activity is also not enough, however. This is despite several studies reporting that most cancer survivors would prefer to receive physical activity counseling or information from a fitness expert or physical activity specialist associated with a cancer center together with input from their health practitioner (i.e., specialist nurse, physician, or oncologist) [21]. To date, the barriers and facilitators to multidisciplinary team members’ routine provision of physical activity information have not been fully studied. Also, the amount of physical activity information that is actually provided by oncology care providers (OCPs) in Japan remains unclear. In order to assess the barriers and facilitators to OCPs systematically providing such information, in this study we used the Consolidated Framework for Implementation Research (CFIR), one of the most common frameworks for guiding systematic research that evaluates the process of implementing the delivery of health care [22–24].

We also considered OCPs’ own high physical activity levels to be one of the facilitators to their routine provision of physical activity information. Some previous studies reported that OCPs with higher physical activity levels themselves tended to have a positive attitude to physical activity promotion and to provide more physical activity information to patients [19, 21, 25], whereas other studies reported no association between OCPs’ own physical activity levels and their provision of physical activity information to patients [26]. Given the conflicting evidence, we examined this as a potential facilitating factor.

The purpose of this study was threefold: 1) to determine OCPs’ awareness of the detailed contents of the JBCS physical activity recommendation, 2) to determine their routine provision of information about the JBCS physical activity recommendation to BCSs, and 3) to reveal the barriers and facilitators related to their awareness and provision.

## Methods

### Participants and procedures

We conducted a web-based self-report questionnaire survey using *SurveyMonkey* (SurveyMonkey, Palo Alto, CA) involving OCPs who were members of the JBCS (*n* = 9,996) or the Japanese Association of Cancer Rehabilitation (JACR, *n* = 834). Between December 2018 and February 2019, we sent an e-mail containing information on the research prospectus and the questionnaire link to each member on the societies’ mailing lists. The prospectus described the study’s purpose and method and the method of consent (checking the “participation” box implied consent to participate), as well as contact information. To avoid duplicate submissions from a member of both societies included in the study, respondents were first asked if they had completed the questionnaire as a member of the other academic society and those who answered affirmatively were automatically restricted from answering further questions. To improve the response rate, we sent three reminders to all members listed. Those who regularly provided medical treatments or care to BCSs for over a 1-year period and were aged ≥ 20 years were included in the analysis. Those who could not respond to the self-reported questionnaire (written in Japanese) or did not answer any questions related to the study outcomes were excluded from the analysis.

### Survey items

A literature review did not identify suitable tools with established reliability and validity for use in this study, so we developed an original questionnaire. We created the questionnaire items based on the results of a focus group interview with OCPs and a review of the literature using the CFIR. We also conducted cognitive checks with multidisciplinary research teams including oncologists who usually provide treatment or care for BCSs, rehabilitation therapists, oncology nurses, fitness trainers, BCSs, exercise physiologists, sports scientists, and psychiatrists.

The questionnaire consisted of 4 sections. Section 1 collected socio-demographic information including age, sex, occupation, whether they were a manager or not, facility background, years of experience in breast cancer care, and frequency of medical care for BCSs. Section 2 asked about awareness and practice related to the JBCS physical activity recommendation, which were the outcome measures. For the question about awareness of the contents of the recommendation, we asked participants to select one of four options that most closely aligned with their experience: “I know its details,” “I may have heard about it, but I don’t know its details,” “I have heard of its existence,” or “I have never heard of it.” For the question about how often they inform BCSs about the benefits of physical activity in practice, they selected one of three options that most closely aligned with their experience: “I routinely explain the benefits of physical activity to BCSs,” “I occasionally explain the benefits of physical activity to BCSs,” or “I have never explained the benefits of physical activity to BCSs before.”

Section 3 asked about possible factors related to awareness and practice (Table 1). We created survey items according to subdomains of the CFIR: 3 items on “intervention characteristics,” 5 on “outer setting,” 10 on “inner setting,” 10 on “characteristics of individuals,” and 3 on “process.”

**Table 1:** Demographic background of the participants and items from the CFIR

		Total	
Demographic Variables		*n*	%
Society member	JBCS	892	86.7
	JACR	137	13.3
Sex	Female	467	45.4
	Male	562	54.6
Age (years)	Mean (standard deviation)	48.0	(9.8)
Occupation	Physician	726	70.6
	Nurse	120	11.7
	Rehabilitation therapist	122	11.9
	Other	61	5.9
Position	Not a manager	480	53.8
	Department manager	311	34.9
	Facility manager	101	11.3
Able to decide about health behavior support efforts for breast cancer patients	Yes	461	55.5
	No	369	44.5
Facility location	Urban	673	65.4
	Rural	356	34.6
Type of facility	Comprehensive cancer center	537	52.2
	Academic medical center	84	8.2
	Community hospital	276	26.8
	Clinic/Home nursing service	98	9.5
	Other	34	3.3
Years of experience in breast cancer treatment (years)	< 5	142	13.8
	≥ 5	887	86.2
Frequency of working on BCS care (times/week)	< 2	128	5.0
	≤ 2, < 5	226	21.9
	≥ 5	675	65.7
Physical Activity Levels			
Exercise habit (per week)	At least once	284	36.6
	Less than once	493	63.4
MVPA (METs-h/day)	Mean (standard deviation)	6.4	(9.2)
OCPs who met the physical activity recommendation	< 60 min/day	337	43.4
	≥ 60 min/day	440	56.6
Sedentary time (h/day)	≤ 3	143	18.4
	< 3, < 8	504	64.9
	≥ 8	130	16.7
"Intervention Characteristics" in the CFIR			
Presence/absence of perception that there is solid evidence for the physical activity recommendation:Do you think there is solid evidence of the association between post-illness physical activity and the risk of death from breast cancer?	I think so	434	52.3
	I don't think so. / I don't know if I think so or not	396	47.7
Presence/absence of perception that helping BCSs to maintain high physical activity levels is time-consuming:Do you think it will take too long to make efforts to help promote physical activity for BCSs?	I strongly think so. / I somewhat think so	562	67.7
	I don't think so at all. / I don't much think so. / I don't know if I think so or not	268	32.3
Presence/absence of perception that helping BCSs to maintain high physical activity is resource-consuming: Do you think it is difficult to make efforts to help promote physical activity for BCSs because it requires a lot of equipment, personnel, and suitable environments?	I strongly think so. / I somewhat think so	475	57.2
	I don't think at all. / I don't much think so. / I don't know if I think so or not	355	42.8
"Outer Setting" in the CFIR			
Presence/absence of awareness that more than 60% of BCSs need help maintaining high physical activity levels: What percentage of BCSs that you care for need explanations about maintaining high physical activity levels?	More than 60% of them need an explanation	617	74.3
	≤ 60% of them need an explanation	213	25.7
Presence/absence of awareness that more than 60% of BCSs are at high risk of health problems from performing MVPA^†^ to maintain high physical activity levels: What percentage of BCSs are at high risk from performing MVPA?	More than 60% of them have health concerns	117	14.1
	≤ 60% have health concerns	713	85.9
Presence/absence of a competing facility for health care delivery in the area: Are there any facilities around your facility that are likely to be competitors?	There are facilities around us with similar functions	471	60.0
	No, there are not such facilities around us	314	40.0
Presence/absence of facilities that provide behavioral support efforts to help BCSs maintain high physical activity levels: Do you know any behavioral support efforts to help BCSs maintain high physical activity levels at other facilities?	I know of such efforts	124	14.9
	I don't know of such efforts	706	85.1
"Inner Setting" in the CFIR			
Presence/absence of the opportunity to discuss survivorship care for BCSs in a multidisciplinary team: Does your facility offer the opportunity to consider survivorship care for BCSs in a multidisciplinary team?	Yes, we do. It's carried out for all survivors or for survivors in need	312	39.7
	No, we have no or few such opportunities	473	60.3
Presence/absence of supportive attitude among managers toward staff efforts to improve evidence-based practice: Do you think the manager of your facility is supportive of staff efforts to improve practice and its structure based on evidence?	I strongly think so. / I somewhat think so	467	63.7
	I don't think so at all. / I don't much think so. / I don't know if. / I think so or not	266	36.3
Presence/absence of experience of reviewing and revising daily practice according to the revised JBCS guidelines: Has your facility revised practice based on the revised version of the JBCS guideline?	As a result of reviewing medical care according to the revised guidelines, the medical care content and system were changed	176	22.4
	Medical care was already in line with the guidelines even before the revision	221	28.2
	We have reviewed medical care and recognize that it needs to be changed, but medical care and the medical care system have not been changed	245	31.2
	There are no plans to review medical care based on the JBCS guideline	143	18.2
Presence/absence of managers' perception that helping BCSs to maintain high physical activity levels is within the scope of service provided at the facility: Do you think the managers of your facility recognize that helping BCSs maintain high physical activity levels is within the scope of service provided at the facility?	I strongly think so. / I somewhat think so	317	43.2
	I don't think so at all. / I don't much think so. / I don't know if. / I think so or not	416	56.8
Presence/absence of any existing resources on behavioral support efforts to help BCSs maintain high physical activity levels: Are there any resources available on behavioral support efforts to help BCSs maintain high physical activity levels?"	Yes, there are	202	25.7
	No, there are not any at all	583	74.3
Presence/absence of material such as a pamphlet providing information about the physical activity recommendation: Are there any materials such as a pamphlet providing information about the physical activity recommendation?	Yes, there is	147	18.7
	No, there is not	638	81.3
Presence/absence of a website providing information about the physical activity recommendation: Is there a website providing information including content related to the physical activity recommendation?	Yes, there is	5	0.6
	No, there is not	780	99.4
Presence/absence of any locations and/or programs at your facility to help BCSs maintain high physical activity levels: Are there any suitable environments and/or programs at your facility that help BCSs maintain high physical activity levels?	Yes, there are	70	8.9
	No, there are not	715	91.1
Presence/absence of opportunities to learn about the revised guideline: Have you ever had the opportunity to learn about the revised contents of the JBCS guideline at your facility?	Yes, we have. / Not so far, but there are plans to do this in the future	206	26.2
	Nothing has happened and there are no plans to do this in the future. / I don't know	579	73.8
Presence/absence of the JBCS guidelines at the facility: Are the JBCS guidelines available at your facility?	The JBCS guidelines are available	484	61.7
	No, they are not available at our facility. / I don't know	301	38.3
"Characteristics of Individuals" in the CFIR			
Presence/absence of experience reading the part about epidemiology and diagnosis in the revised guidelines: Have you ever read the part about epidemiology and diagnosis in the revised JBCS guidelines?	Yes	715	69.5
	No	314	30.5
Presence/absence of awareness that helps BCSs to maintain high physical activity levels is within the scope of work in the respondents' profession: Is your occupation responsible for explaining the need to maintain high physical activity levels in your facility?	Yes	764	80.7
	No	183	19.3
Presence/absence of accurate knowledge of the guideline recommendation: Do you think it is recommended that BCSs maintain high physical activity levels?	I think so	752	90.6
	I don't think so. / I don't know	78	9.4
Presence/absence of accurate knowledge of the recommended intensity of physical activity for BCSs: What is the recommended intensity of physical activity for BCSs?	Medium intensity / Vigorous-intensity	626	75.4
	Low intensity / I don't know	204	24.6
Presence/absence of motivation to provide the physical activity recommendation: Do you want to inform BCSs of the physical activity recommendation so that they can maintain high physical activity levels?	I strongly want to. / I somewhat want to	756	91.1
	I don't want to at all. / I don't much want to. / I don't know if. / I want to or not	74	8.9
Presence/absence of awareness that helping BCSs maintain high physical activity levels is within the scope of service provided at the facility: Do you think that providing information on maintaining physical activity should be addressed at your facility?	I strongly think so. / I somewhat think so	732	88.2
	I don't think so at all. / I don't much think so. / I don't know if. / I think so or not	98	11.8
Presence/absence of awareness that helping BCSs to maintain high physical activity levels is within the scope of the respondents' work responsibilities: Do you think it's your role to explain to BCSs the need to maintain high physical activity levels?	I think it's part of my role	556	67.0
	I don't think it's my role	274	33.0
Presence/absence of willingness to take leadership in helping BCSs maintain high physical activity levels: Are you willing to take leadership at your facility for behavioral support initiatives that help BCSs maintain high physical activity levels?	I am very willing. / I am somewhat willing	515	62.0
	I am not at all willing. / I am not that willing. / I don't know if I am willing or not	315	38.0
Presence/absence of self-efficacy in terms of informing BCSs about the physical activity recommendation: Are you confident in informing BCSs about the physical activity recommendation?	I am very confident. / I am somwehat confident	393	47.3
	I am not confident at all. / I am not very confident. / I don't know if I am confident or not	437	52.7
Presence/absence of additional learning needs for informing about the physical activity recommendation:Do you think you need to participate in additional training and study sessions to deliver the physical activity recommendation?	I strongly think so. / I somewhat think so	618	74.5
	I don't think so at all. / I don't much think so. / I don't know if I think so or not	212	25.5
"Process" in the CFIR			
Presence/absence of outpatient nursing counseling services: Does your facility have an outpatient nursing counseling service for BCSs?	Yes, it does. We have it, including support for BCSs to maintain high physical activity levels	113	14.4
	Yes, it does. We have it, but it does not include support for BSCs to maintain high physical activity levels	224	28.5
	No, we do not have such a service	448	57.1
Presence/absence of outpatient rehabilitation services: Does your facility have outpatient rehabilitation services for BCSs?	Yes, it does. We have them, including support for BCSs to maintain high physical activity levels	126	16.1
	Yes, it does. We have them, but they do not include support for breast cancer survivors to maintain high physical activity levels	398	50.7
	No, we do not have such services	261	33.2
Presence/absence of organizational culture that regularly reviews daily operations and systems for quality improvement: Does your facility regularly check daily operations and systems to improve the quality of care provided?	I strongly think so. / I somewhat think so	484	61.7
	I don't think so at all. / I don't much think so. / I don't know if. / I think so or not	301	38.3

*BCS* breast cancer survivor, *CFIR* Consolidated Framework for Implementation Research, *JACR* Japanese Association of Cancer Rehabilitation, *JBCS* Japan Breast Cancer Survivor, *MVPA* moderate-to-vigorous physical activity

Section 4 asked participants about their own physical activity levels using the Japan Public Health Center-based prospective study-physical activity questionnaire-short form (JPHC-PAQ-Short) [27], which has been validated [27, 28]. This 3-item scale consist of “heavy physical work or strenuous exercise” (“none,” “under 1 h,” and “1 h or more”), “walking and standing” (“under 1 h,” “1–2 h,” and “3 h or more”), and “sedentary activity” (“3 h or less,” “3–8 h,” and “8 h or more”). OCPs’ physical activity levels were assessed by calculating the amount of moderate-to-vigorous physical activity (MVPA) according to the scale manual [27, 28] and whether these met the physical activity level recommended by Japan’s Ministry of Health, Labour and Welfare [29]. We also asked participants to select 1 of 5 response options about their exercise habits in leisure time (“almost none” to “almost every day”).

### Data analysis

We calculated descriptive statistics for OCPs’ awareness and practice related to the JBCS physical activity recommendation. Awareness of the recommendation was assessed as a binary variable according to whether or not they were aware of the details. The practice of providing the recommendation was assessed as a binary variable according to whether or not they regularly explained the benefits of physical activity to BCSs. To identify possible factors related to awareness and practice, we conducted logistic regression analysis with a backward elimination technique using each of the two dichotomous variables of awareness and practice as an outcome variable and with all relevant variables as explanatory variables.

All tests were two-tailed with a *p* value < 0.05 indicating statistical significance. All statistical analyses were performed using SAS® Ver.9.4 (SAS Institute Inc., Cary, NC).

## Results

**Fig. 1:**
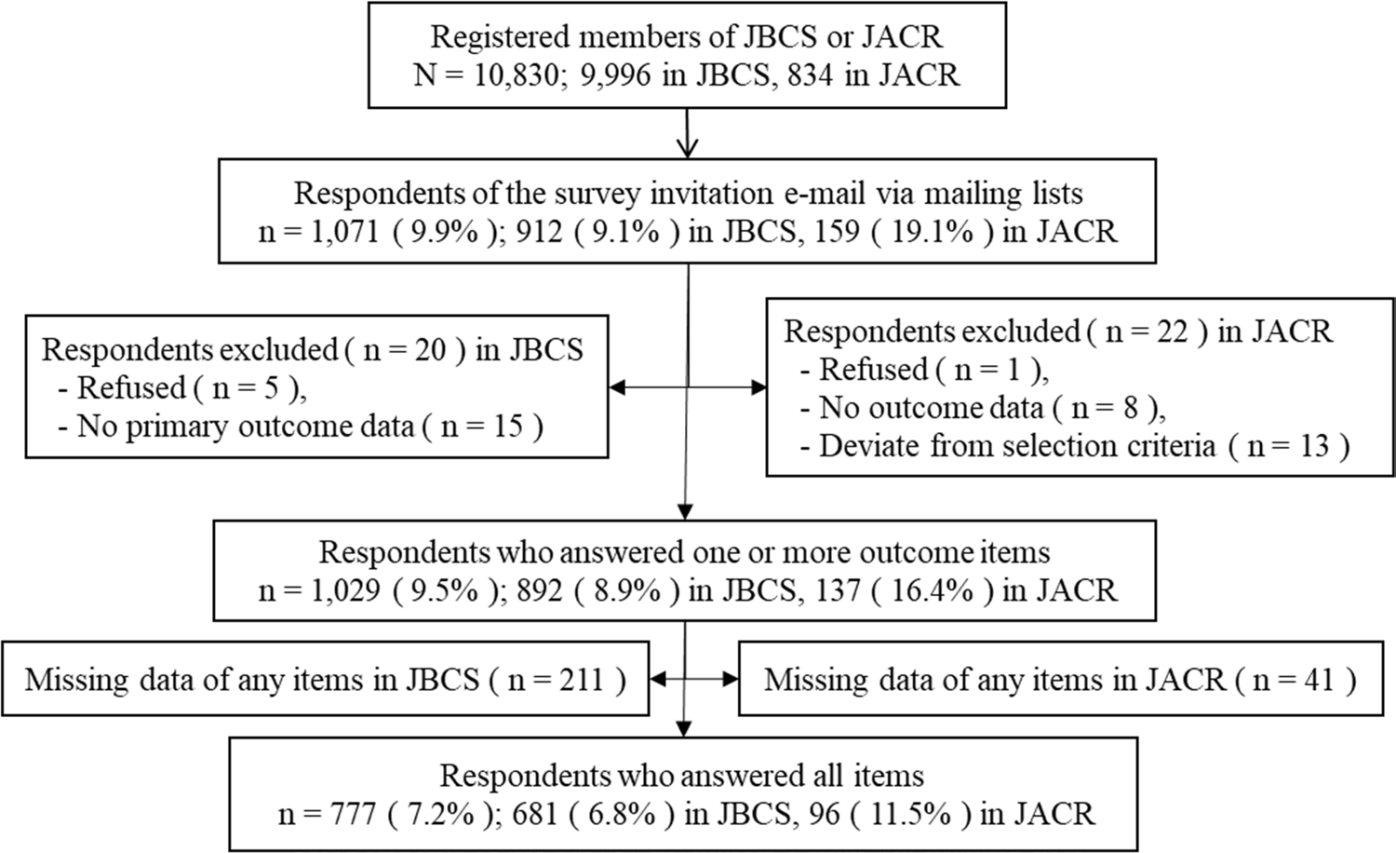
Participants’ recruiting flow diagram. JBCS: Japanese Breast Cancer Society, JACR: Japanese Association of Cancer Rehabilitation

Among the members of the JBCS, 912 (9.1%) responded to the survey and 892 (8.9%) answered the questions about awareness and practice related to the physical activity recommendation. The corresponding numbers for the JACR were 159 (19.1%) and 137 (16.4%). The total number of participants included in the analysis was 1029 (9.5%) (Fig. 1).

### Participant characteristics

Participant characteristics are summarized in Table 1. Overall, 54.6% of participants were male and the mean age was 48.0 (standard deviation: 9.8) years. The occupation of most of the participants was physician (70.6%), followed by nurse (11.7%) and rehabilitation therapist (11.9%). Most had experience caring for BCSs for 5 years or more and were caring for BCSs 5 or more times per week.

### Participants’ awareness and practice related to the JBCS physical activity recommendation

**Fig. 2:**
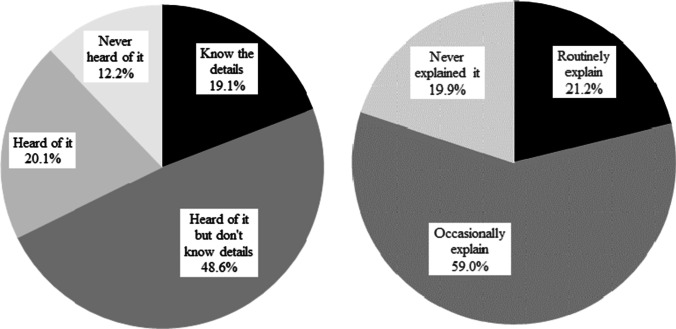
Percentage of participants who were aware of and explained the contents of the physical activity recommendation in the Japan Breast Cancer Society Clinical Practice Guidelines. a. Percentage of participants who knew the contents of the physical activity recommendation in the JBCS guidelines. b. Percentage of participants who routinely explain the benefits of maintaining high physical activity levels to breast cancer survivors

Only 19.1% of participants knew the details of the JBCS physical activity recommendation, 48.6% had heard of it but did not know the details, and 20.1% had heard of it. In addition, only 21.2% routinely explained the benefits of physical activity to BCSs and 59.0% occasionally explained it (Fig. 2).

### Items created using the CFIR

#### Intervention characteristics

Approximately half of the participants believed there was insufficient evidence for the effects of physical activity on breast cancer-related outcomes. Many participants felt efforts to promote physical exercise to BCSs to help them maintain high physical activity levels were too time-consuming and resource-consuming (Table 1).

#### Outer setting

Many participants (75%) recognized that more than 60% of BCSs needed support around physical activity.　Few participants (14%) believed that more than 60% of BCSs are at high risk of health problems from performing MVPA. Only 9.8% of the participants reported that their facilities work with other facilities to help BCSs maintain high physical activity levels. Only 15% knew of any behavioral support efforts to maintain high physical activity for BCSs at other facilities (Table 1).

#### Inner setting

Approximately 40% of the participants had opportunities to consider survivorship care, including helping BCSs maintain high physical activity, in a multidisciplinary team. Half of them had experience revising their daily practice in accordance with the revised JBCS guidelines or had confirmed their practice was in line with the revised guidelines. Only one-quarter of them had opportunities to learn about the revised guidelines. Regarding the current resources available for BCSs to maintain physical activity levels at their facilities, 19% reported having some material such as a pamphlet providing information about the physical activity recommendation, 0.6% had a website providing information about the physical activity recommendation, and only 8.9% reported having any suitable environments or programs at their facility. Approximately 60% reported that the JBCS guidelines were available at their facility (Table 1).

#### Characteristics of participants

Approximately 70% had read the part about epidemiology and diagnosis in the revised JBCS guidelines. Overall, 91% wanted to inform BCSs so that they could maintain high physical activity levels, and 80% recognized that explaining the need to maintain high physical activity levels was within the scope of work in their profession and also within the scope of services provided at their facilities. Approximately 60% were willing to take on leadership roles at their facilities for behavioral support initiatives to help BCSs maintain the recommended physical activity levels.

Approximately 90% knew that it is recommended that BCSs maintain high physical activity levels. On the other hand, 25% did not know the recommended exercise intensity. Half of the respondents were not confident in informing BCSs about the physical activity recommendation and 75% had additional learning needs about the recommendation (Table 1).

#### Process

Only 14.4% of participants reported that their facilities had outpatient nursing counseling services that included support for BCSs to maintain high physical activity levels versus 29% who reported that their facilities had outpatient nursing counseling services that did not include such support. Although 68% reported their facilities had outpatient rehabilitation services, only 16% reported that these included such support.

#### Barriers and facilitators to participants’ awareness and practice related to the physical activity recommendation

The results of multivariate analysis (Table 2) showed that participants who tended to routinely explain the recommendation were characterized as follows: 1) perceived that helping BCSs to maintain high physical activity levels was within the scope of their profession’s work (odds ratio [OR]: 6.1, *p* < 0.01); 2) perceived that more than 60% of BCSs need help maintaining high physical activity levels (OR: 2.4, *p* < 0.01); 3) were rehabilitation therapists (OR:2.2) or nurses (OR:1.7) (*p* = 0.03); 4) worked at facilities that had outpatient nursing counseling services that included support for BSCs to maintain high physical activity levels (OR: 2.1) or did not include such support (OR: 1.3) (p = 0.03); 5) knew the details of the physical activity recommendation (OR: 1.8, *p* = 0.01); 6) perceived that there was solid evidence for the physical activity recommendation (OR: 1.6, *p* = 0.03); 7) worked at facilities that had any suitable environment or program to help BCSs maintain high physical activity levels (OR: 1.9, *p* = 0.03); 8) had self-efficacy in terms of informing BCSs about the physical activity recommendation (OR: 1.6, *p* = 0.03); 9) had accurate knowledge of the recommended level of physical activity (OR: 1.7, *p* = 0.03); 10) met the physical activity recommendations themselves (OR: 1.6, *p* = 0.04); and 11) perceived that helping BCSs to maintain high physical activity levels is within the scope of their work responsibilities (OR: 1.7, *p* = 0.04).

**Table 2:** Barriers and facilitators to oncology care providers’ routinely explaining the physical activity recommendation in the JBCS revised guidelines

Explanatory variables		OR	95% CI	*p*
Presence/absence of awareness that helps BCSs to maintain high physical activity levels is within the scope of work in the respondents' profession	Presence	6.1	2.71–16.44	< 0.001
	Absence		ref	
Presence/absence of awareness that more than 60% of BCSs need help maintaining high physical activity levels	Presence	2.4	1.46–4.20	0.001
	Absence		ref	
Occupation	Physician		ref	0.031
	Nurse	1.7	0.89–3.01	
	Rehabilitation therapist	2.2	1.24–3.80	
	Other	1.0	0.27–3.40	
Presence/absence of outpatient nursing counseling services	Presence of outpatient nursing counseling services, including support for maintaining high physical activity for breast cancer survivors	2.1	1.22–3.53	0.026
	Presence of outpatient nursing counseling services, not including support for maintaining high physical activity for breast cancer survivors	1.3	0.82–1.93	
	Absence of outpatient nursing counseling services		ref	
Presence/absence of awareness about the details of the physical activity recommendation	Presence	1.8	1.14–2.80	0.011
	Absence		ref	
Presence/absence of perception that there is solid evidence for the physical activity recommendation	Presence	1.6	1.06–2.45	0.026
	Absence		ref	
Presence/absence of any locations and/or programs at your facility to help BCSs maintain high physical activity levels	Presence	1.9	1.05–3.52	0.032
	Absence		ref	
Presence/absence of self-efficacy in terms of informing BCSs about the physical activity recommendation	Presence	1.6	1.05–2.39	0.029
	Absence		ref	
Presence/absence of accurate knowledge of the recommended intensity of physical activity for BCSs	Presence	1.7	1.06–2.95	0.033
	Absence		ref	
OCPs who met the physical activity recommendations	< 60 min/day	1.6	1.02–2.48	0.039
	≥ 60 min/day		ref	
Presence/absence of awareness that helping BCSs to maintain high physical activity levels is within the scope of the respondents' work responsibilities	Presence	1.7	1.03–2.86	0.041
	Absence		ref	

Logistic regression analysis with backward elimination (*p* < 0.05); *R*2 = 0.17, modified *R*2 = 0.26 c statistics = 0.78 *JBCS* Japan Breast Cancer Survivor, *JACR* Japanese Association of Cancer Rehabilitation, *BCS* Breast Cancer Survivor, *CFIR* Consolidated Framework for Implementation Research, *MVPA* Moderate to vigorous physical activity, *OR* odds ratio, *Ci* confidence interval, *ref* reference

The following participants tended to know the details of the physical activity recommendation (Table 3): 1) had already read the part about epidemiology and diagnosis in the revised guidelines (OR: 7.4, *p* < 0.01); 2) had self-efficacy in terms of informing BCSs about the physical activity recommendation (OR: 2.9, *p* < 0.01); 3) perceived that helping BCSs to maintain high physical activity levels was within the scope of work in their profession (OR: 3.1, *p* < 0.01); 4) worked at facilities that had outpatient nursing counseling services that included support BCSs to maintain high physical activity levels (OR: 2.0) or did not include such support (OR: 1.05) (*p* = 0.04); 5) perceived that there was solid evidence for the physical activity recommendation (OR: 1.8, *p* = 0.01); 6) had the opportunity to discuss survivorship care for BCSs in a multidisciplinary team (OR: 1.6, *p* = 0.02); 7) worked at facilities where the JBCS guidelines were available (OR: 1.7, *p* = 0.04); and 8) had accurate knowledge of the recommended levels of physical activity (OR: 1.8, *p* = 0.048).

**Table 3:** Barriers and facilitators to oncology care providers' awareness of the details of the physical activity recommendation of the JBCS revised guidelines

Explanatory variables		Ors	95% Cis	*p*
Presence/absence of experience reading the part about epidemiology and diagnosis in the revised guidelines	Presence	7.4	3.52 – 18.2	< .0001
	Absence		ref	
Presence/absence of self-efficacy in terms of informing BCSs about the physical activity recommendation	Presence	2.9	1.88—4.59	< .0001
	Absence		ref	
Presence/absence of awareness that helps BCSs to maintain high physical activity levels is within the scope of work in the respondents' profession	Presence	3.1	1.47 – 7.39	0.005
	Absence		ref	
Presence/absence of perception that there is solid evidence for the physical activity recommendation	Presence	1.8	1.13 – 2.88	0.015
	Absence		ref	
Presence/absence of the opportunity to discuss survivorship care for BCSs in a multidisciplinary team	Presence	1.6	1.07 – 2.47	0.023
	Absence		ref	
Presence/absence of the JBCS guidelines at the facility	Presence	1.7	1.04 – 2.88	0.037
	Absence		ref	
Presence/absence of outpatient nursing counseling services	Presence of outpatient nursing counseling services, including support for maintaining high physical activity for breast cancer survivors	2.0	1.14 – 3.57	0.042
	Presence of outpatient nursing counseling services, not including support for maintaining high physical activity for breast cancer survivors	1.05	0.64 – 1.68	
	Absence of outpatient nursing counseling services		ref	
Presence/absence of accurate knowledge of the recommended intensity of physical activity for BCSs	Presence	1.8	1.02 – 3.26	0.048
	Absence		ref	

Logistic regression analysis with backward elimination (*p* < 0.05). *R*2 = 0.17, modified *R*2 = 0.28, c statistics = 0.81. *JBCS* Japan Breast Cancer Survivor, *JACR* Japanese Association of Cancer Rehabilitation, *BCS* Breast Cancer Survivor, *CFIR* Consolidated Framework for Implementation Research, *MVPA* Moderate to vigorous physical activity, *OR* odds ratio, *Ci* confidence interval, *ref* reference

## Discussion

This is the first study to clarify OCPs’ awareness and practice related to the physical activity recommendation for BCSs in the JBCS guidelines. Even though cancer survivors show a strong preference for receiving information about exercise behavior from their oncologist [14, 15], only 21.2% of the OCPs in the present study routinely tell BCSs about the physical activity recommendation.

Among the OCPs in this study, their perception about the scope of work responsibilities in their own profession was one of the most significant related factors in deciding whether or not to routinely explain the physical activity recommendation to BCSs. Notably, those who thought provision of physical activity information was not within the scope of work in their profession or within the scope of their own work responsibilities at their facility did not implement the recommendation. Therefore, an approach is needed that recognizes the provision of physical activity information as a routine part of care for BCSs.

OCPs who did not recognize that more than 60% of BCSs need help maintaining high physical activity levels tended not to routinely explain the physical activity recommendation. We chose to use the proportion of 60% in our questionnaire item given that 54–67% of American BCSs do not meet the recommended physical activity levels [8–10]. We used overseas data in this case because there are no such data available for Japan other than currently unpublished data in a study that we conducted and will report on shortly [30]. Clearly, it is important to inform OCPs that there are many BCSs who potentially need help in maintaining high physical activity levels because they do not meet the JBCS recommendation.

OCPs who worked at facilities with available resources tended to routinely explain the physical activity recommendation more than those who worked at facilities without such resources. On the other hand, 68% perceived provision of the physical activity recommendation as time-consuming and 57% perceived it as resource-consuming. Thus, lack of time and available resources are barriers to OCPs routinely explaining the recommendation. This result is similar to that of previous studies [19, 21]. Rehabilitation therapists who responded to our questionnaire tended to explain about the physical activity recommendation more than other OCPs, yet only 16% of their institutions had outpatient rehabilitation services to help BCSs maintain high physical activity levels. Also, 29% of our respondents reported that their facilities had outpatient nursing counseling services that did not include support for BCSs to maintain high physical activity levels and 51% reported that their facilities had outpatient rehabilitation services that did not include such support. There might be potential for these facilities to offer outpatient nursing and rehabilitation services that include such support. As only 9.8% of respondents answered that they are currently cooperating with other facilities in the area, promoting such collaboration may be an option to compensate for the lack of available resources. Proposing specific exercise programs to BCSs will likely be difficult in practice due to limited time and resources. To help address this, we have developed a home-based exercise program for BCSs that does not require the use of special tools and can be completed in a short time [31, 32].

In this study, self-efficacy was one of the most significant facilitators in the routine provision of the physical activity recommendation. This is consistent with the suggestion by Hardcastle et al. that increasing OCPs’ confidence in physical activity promotion may improve their physical activity promotion behavior [25]. However, approximately half of our respondents did not have self-efficacy and around 75% wanted additional training and study sessions. We suggest that all OCPs be told specifically about the recommendation, so as to provide them the necessary knowledge and skills to implement the recommendation and thereby improve efficacy expectation [33, 34]. On the other hand, it is also important to consider the outcome expectation. About half of respondents thought there was insufficient evidence for the physical activity recommendation. In the free description section of our questionnaire, some OCPs stated that it was not clear what kind of physical activity should be specifically recommended. Both in this study and a previous study by Park et al. [19], barriers to recommending exercise for cancer survivors were concerns about the effectiveness of exercise and perceived unclear recommendations. Because it is not clear exactly what types of physical activity programs are efficacious and efficient [19, 35], further research is warranted. Furthermore, most of the evidence available on physical activity originates from Europe and the USA [1–5], and as far as we know, there have been no previous studies concerning Japanese BCSs. It is known that physique and lifestyle, including physical activity levels, differ between Japanese and Western populations [36], and there are also racial differences in the risk of developing breast cancer [37] and in the outcomes for breast cancer [38]. Therefore, further research involving Japanese BCSs is needed.

Factors related to provision of the physical activity recommendation were awareness of its details and accurate knowledge of the recommended physical activity levels. This is similar to a previous finding that one of the most significant barriers to exercise discussion was OCPs’ insufficient knowledge [21]. Factors related to being aware of the details of the physical activity recommendation were the same as some of the related factors for provision of the recommendation. Besides these common factors, the related factors of being aware of the recommendation details were 1) availability of the JBCS guidelines at the facility, 2) experience reading the relevant part in the revised guidelines, and 3) opportunity to discuss survivorship care in a multidisciplinary team. Given related factor 2), it will be important to promote the revised guidelines in collaboration with academic societies. Discussing survivorship care in a multidisciplinary team is thought to encourage understanding of the relevant evidence and guidelines, and we suggest offering OCPs such opportunities. Furthermore, bivariate analysis showed that experience of reviewing and revising daily practice in accordance with the revised JBCS guidelines tended to be a facilitator to awareness of the recommendation details (Table 3), so we also recommend creating a culture that confirms daily practice based on the evidence provided in the guidelines.

OCPs’ own high physical activity levels were one of the facilitators in the routine provision of the physical activity recommendation. This finding is consistent with that of previous studiers [19, 21, 25] where OCPs with higher physical activity levels themselves tended to have positive attitudes to physical activity promotion and provide more physical activity information to patients. Compared with more than 60% of OCPs not meeting the physical activity recommendations themselves in previous studies [26], our OCPs had higher physical activity levels (57% of OCPs met the physical activity recommendation), and it is possible that OCPs with a more positive attitude toward physical activity promotion responded, overestimating the impact. Therefore, further research is needed to confirm whether own high physical activity level is a facilitator in the routine provision of the physical activity recommendation.

There were several limitations in this study. The results cannot be generalized to all OCPs. There could have been participation bias because the response rates were low (JBCS: 8.9% and JACR: 16.4%) and those who volunteered to participate might have had a strong interest in the physical activity recommendation.

## Conclusion

We clarified that only one fifth of OCPs routinely provide physical activity information and only 19.1% of them are aware of the detailed contents of the JBCS physical activity recommendation for BCSs in Japan. Barriers to their routine provision of the physical activity recommendation were 1) perception that the recommendation was not within the scope of their work responsibilities, 2) underestimation of survivors’ physical activity needs, 3) lack of resources, 4) lack of self-efficacy, and 5) poor knowledge of the recommendation. In addition, the related factors of being aware of the details of the recommendation were 1) availability of the JBCS guidelines at the facility, 2) experience reading the part about epidemiology and diagnosis in the guidelines, and 3) opportunity to discuss survivorship care for survivors in a multidisciplinary team.

Thus, to facilitate implementation of the provision of the physical activity recommendation, we suggest the following actions: 1) disseminate the JBCS revised guidelines to all OCPs, 2) provide education and training programs for OCPs about promoting physical activity, 3) develop institutional resources and/or strengthen collaboration with surrounding resources to help maintain high physical activity levels in BCSs, 4) conduct further research to confirm the benefits of physical activity for BCSs in the Japanese population, and 5) develop programs to help BCSs maintain high physical activity levels that are less costly in terms of time and resources than those currently available. In addition, conducting case meetings in a multidisciplinary team and reviewing evidence-based clinical practice will enhance dissemination and implementation of the guidelines.

## Supplementary Information

Below is the link to the electronic supplementary material.

Supplementary file1 (DOCX 48 KB)

## Data Availability

The data that support the findings of this study are available from the corresponding author upon reasonable request.
