# Supplementary material for: Oncology care providers’ awareness and practice related to physical activity promotion for breast cancer survivors and barriers and facilitators to such promotion: a nationwide cross-sectional web-based survey
Source: Support Care Cancer. 2021 Dec 1;30(4):3105–18. doi: 10.1007/s00520-021-06706-8 (PMC8857119; doi:10.1007/s00520-021-06706-8)
Supplement: Supplementary file 1 — Supplementary file1 (DOCX 48 KB) [file 520_2021_6706_MOESM1_ESM.docx]

Table S1. Discriptive data on factors related to oncology care providers' awareness and provision with the physical activity recommendation in the JBCS revised guidelines
